# Editorial: The Molecular Mechanisms of Synaptic Plasticity Impairments in Alzheimer’s Disease

**DOI:** 10.3389/fcell.2022.832728

**Published:** 2022-01-21

**Authors:** Gong-Ping Liu, Peng Lei, Zhi-Fang Dong, Shu-Peng Li

**Affiliations:** ^1^ Key Laboratory of Ministry of Education of China and Hubei Province for Neurological Disorders, Department of Pathophysiology, School of Basic Medicine, The Collaborative Innovation Center for Brain Science, Tongji Medical College, Huazhong University of Science and Technology, Wuhan, China; ^2^ Co-Innovation Center of Neuroregeneration, Nantong University, Nantong, China; ^3^ Department of Neurology and State Key Laboratory of Biotherapy, West China Hospital, Sichuan University, Chengdu, China; ^4^ Ministry of Education Key Laboratory of Child Development and Disorders, Chongqing Key Laboratory of Translational Medical Research in Cognitive Development and Learning and Memory Disorders, National Clinical Research Center for Child Health and Disorders, Children’s Hospital of Chongqing Medical University, Chongqing, China; ^5^ School of Chemical Biology and Biotechnology, Peking University Shenzhen Graduate School, Shenzhen, China

**Keywords:** Alzheimer’s disease, synaptic plasticity, spine, dendritic plasticity, cognition

Synaptic plasticity, which is directly related to memory and learning processes, is determined by morphological and functional modifications of synapses. Long-term potentiation and long-term depression are two main manifested forms of synaptic plasticity. Dendritic spines are small, highly dynamic protruding structures in the dendritic membrane, which have specialized subdomains with specific functions in synaptic transmission and plasticity, including scaffolding proteins, ion channels, cytoskeleton components, signal transduction molecules, and postsynaptic density (a complex mainly consisted by AMPAR and NMDAR) (Saneyoshi et al., 2010; González-Burgos, 2012). Structural changes, such as spines elongation and contraction, shape variations, spine distribution/density and functions mediate synaptic plasticity (Chidambaram et al., 2019). Impairments of synaptic plasticity, such as spine shape and density alteration, lead to synaptic dysfunction and cognitive impairment, which is found in many neurodegenerative diseases, including Alzheimer’s disease (AD). Aberrant synaptic structure/morphology and decreased spine density in the hippocampus and neocortex is an early event and a major change, correlated with cognitive deficits in AD (Scheff et al., 1990; Scheff and Price, 2003), which usually appear well before neuronal loss. However, questions about the details and mechanisms of synaptic plasticity dysfunction in AD still warrant further studies, as in contrast with the well documented synaptic dysfunction elicited by pathological factors of accumulated phosphorylated tau and amyloid β peptide (Aβ). This topic focused on “*The Molecular Mechanisms of Synaptic Plasticity Impairments in Alzheimer’s Disease*,” and there were 21 manuscripts to be expected, 13 manuscripts actually submitted and 10 manuscripts accepted.

In this issue of frontiers, Mahaman et al. reviewed the role of STriatal-Enriched protein tyrosine phosphatase (STEP) in dendritic plasticity impairments in AD, whose level and activity are increased in AD *via* Aβ. STEP dephosphorylated and inactivated synaptic proteins *via* kinases such as Fyn, Pyk2 and ERK1/2 (Venkitaramani et al., 2009; Li et al., 2014), and it also led to the internalization of synaptic receptor complexes like GluN2B/GluN1 and GluA2/GluA1 subunits of NMDA and AMPA receptors, respectively (Poddar et al., 2010; Zhang et al., 2010). Furthermore, STEP dephosphorylated SPIN90 to dissociate from cofilin, and activated cofilin to depolymerize F-actin to G-actin. Together, increase of STEP led to synapse loss and dendritic plasticity impairments, and ultimately, resulting in cognitive deficits in AD. Transmembrane protein 59 (TMEM59) reported to associate with AD was introduced by Meng et al. They showed that TMEM59 haploinsufficiency rescued memory defects and synaptic plasticity dysfunction in 5×FAD mice, which overexpressing human APP and PSEN1 transgenes with a total of five AD-linked mutations: the Swedish (K670N/M671L), Florida (I716V), and London (V717I) mutations in APP, as well as M146L and L286V mutations in PSEN1, and rapidly developing severe amyloid pathology and presenting synaptic degeneration. The authors found that overexpression of TMEM59 in the hippocampus caused memory deficits and had a trend to induce synaptic plasticity impairment in wild-type mice, which suggesting its neurotoxic role. Interestingly, while TMEM59 overexpression had no effect on worsening synaptic defects and impaired memory in the 5×FAD mouse model of AD, though it significantly exacerbated AD-like pathologies by increasing levels of detergent-insoluble Aβ and Aβ plaques. They proposed that, due to a mild impact on cognitive and synaptic function impairments, overexpressing TMEM59 may not be able to further worsen the quickly degenerative phenotypes in 5×FAD mice.

As one of the main pathophysiologic markers in AD, hyperphosphorylated tau led to the dissociation of Tau/Fyn/PSD-95/NMDAR complex, the disruption of synaptic potentiation required for LTP (Frandemiche et al., 2014), and the reduction of functional dendritic spine number (Tracy and Gan, 2018). Overexpressing tau decreased NMDAR level by activation of STAT1 and inactivation of STAT3 (Li et al., 2019; Hong et al., 2020). Jiang et al. found that, PINK1 overexpression ameliorated the decreased dendritic spine density *via* autophagy activation to decrease total and phosphorylated tau. In addition to abnormal aggregated tau or Aβ, multiple other risk factors, such as aging, oxidative stress, calcium signal dysregulation, neuroinflammation, genetic and environmental factors, could induce synaptic plasticity defects. In this issue of frontier, a study demonstrated that maternal lead (Pb) exposure induced synaptic plasticity impairment of offspring by reducing GLUT4 protein level in the cell membrane as well as glucose uptake via the PI3K-Akt signaling pathway.

Mitochondrial dysfunction is a well-established early etiological event in AD, which decreases ATP production, changes cytoplasmic calcium concentrations, and increases ROS/NO production, consequently leading to synaptic plasticity abnormalities. Huang et al. and Chen et al., respectively, found that, tetramethylpyrazine or dauricine, the extract from the rootstock of traditional Chinese medicine, modified the mitochondrial protein profile of AD animal models to increase ATP production and some synapse-related protein expression, and ultimately, improved synaptic plasticity and cognition.

Most of the accepted articles in this research topic contributed to expand and deepen our understanding of the risk factors and its mechanisms for synaptic plasticity damage in AD, and we understand that gestational Pb exposure could induce synaptic plasticity impairment of offspring, and etramethylpyrazine and dauricine ameliorate synaptic plasticity, which both are not reported previously. However, some questions, which we have a lot of interest in, are missing in this topic. For example, is LTP impairment caused by directly reduced synapse numbers or decreased synaptic transmission efficiency? What are the inherent molecular mechanisms underlying the aberrant spine number loss (synaptic-associated protein loss, microglia-mediated synapse elimination, neuroinflammation, neuron apoptosis or death)? We hope that the articles in this topic will be of interest to a broad range of researchers working in dendritic plasticity in AD, stimulating experimental work relating to the mechanisms of synaptic plasticity impairments and therapeutic strategies. In a future topic collection, we would like to see more articles about new molecular strategies directly targeting to promote synaptic plasticity.
